# The longitudinal study of subjective wellbeing and absenteeism of healthcare workers considering post-COVID condition and the COVID-19 pandemic toll

**DOI:** 10.1038/s41598-023-37568-1

**Published:** 2023-07-04

**Authors:** Mayssam Nehme, Laure Vieux, Laurent Kaiser, François Chappuis, Catherine Chenaud, HealthCo Study Team, Olivia Braillard, Delphine S. Courvoisier, Jean-Luc Reny, Frederic Assal, Guido Bondolfi, Christophe Graf, Dina Zekry, Silvia Stringhini, Hervé Spechbach, Frederique Jacquerioz, Julien Salamun, Frederic Lador, Ivan Guerreiro, Matteo Coen, Thomas Agoritsas, Lamyae Benzakour, Stéphane Genevay, Kim Lauper, Philippe Meyer, Nana Kwabena Poku, Basile N. Landis, Marwène Grira, Gilles Allali, Pauline Vetter, Idris Guessous

**Affiliations:** 1 Division of Primary Care Medicine, Geneva University Hospitals, Geneva, Switzerland; 2 Division of Occupational Medicine, Geneva University Hospital, Geneva, Switzerland; 3 Faculty of Medicine, University of Geneva, Geneva, Switzerland; 4 Division of Infectious Diseases, Geneva University Hospitals, Geneva, Switzerland; 5 Geneva Center for Emerging Viral Diseases, Geneva University Hospitals, Geneva, Switzerland; 6 Division of Laboratory Medicine, Laboratory of Virology, Geneva University Hospitals, Geneva, Switzerland; 7 Division of Tropical and Humanitarian Medicine, Geneva University Hospitals, Geneva, Switzerland; 8 Quality of Care Division, Medical Directorate, Geneva University Hospitals, Geneva, Switzerland; 9 Division of General Internal Medicine, Geneva University Hospitals, Geneva, Switzerland; 10 Division of Neurology, Geneva University Hospitals, Geneva, Switzerland; 11 Division of Psychiatry, Geneva University Hospitals, Geneva, Switzerland; 12 Department of Rehabilitation and Geriatrics, Geneva University Hospitals, Geneva, Switzerland; 13 Division of Pulmonary Medicine, Geneva University Hospitals, Geneva, Switzerland; 14 Division of Rheumatology, Geneva University Hospitals, Geneva, Switzerland; 15 Division of Cardiology, Geneva University Hospitals, Geneva, Switzerland; 16 Division of Otolaryngology, Geneva University Hospitals, Geneva, Switzerland; 17 Leenaards Memory Center, Lausanne University Hospital, Lausanne, Switzerland

**Keywords:** Occupational health, Health care

## Abstract

Experts have warned against the pandemic burden on healthcare workers early on, however little is known about the evolution of this burden with time, in addition to the long-term effects of post-COVID symptoms in healthcare workers. Staff at the Geneva University Hospitals in Switzerland had an online follow-up in July and December 2021, on their physical and mental health, quality of life and functional capacity using validated scales. Descriptive analyses compared the prevalence of symptoms, functional impairment and quality of life in SARS-CoV-2 positive and negative individuals at baseline and at follow-up. Out of the initial n = 3,083 participants that answered at baseline in July 2021, n = 900 (mean age of 46.4 years, 70.1% women) completed the follow-up in December 2021. With time, more individuals reported fatigue (+ 9.4%), headache (+ 9.0%), insomnia (+ 2.3%), cognitive impairment (+ 1.4%), stress/burnout (+ 8.8%), pain (+ 8.3%), digestive symptoms (+ 3.6%), dyspnea (+ 1.0%), and cough (+ 7.7%) compared to baseline, with a differentially larger increase in symptoms in the SARS-CoV-2 negative group. Individuals had more functional impairment (12.7% at baseline and 23.9% at follow-up), with more absenteeism and worsening quality of life. Healthcare workers are potentially suffering from long term consequences of the pandemic burden, calling for urgent action and solutions.

The healthcare profession has long been one of the most stressful professions with healthcare workers having to deal on a daily basis with decision-making situations that can have a serious impact^1^. Factors such as shift schedules, complexity of patients and situations, as well as having to make timely and important decisions on a day-to-day basis have been shown to contribute to intense work conditions^1,2^. Under usual working conditions, burnout is detected in healthcare professionals who are at a higher risk of anxiety, depression, sleep disorders and post-traumatic stress disorder^3,4^. The COVID-19 pandemic has additionally brought extra stress on a system that might have already been stretched out and at a high risk of burnout^3,4^. The COVID-19 pandemic might have long-term effects on healthcare workers as had been previously reported with the SARS epidemic of 2003^5^, and this time on a larger scale and a more protracted time course^6^.

Early on, studies and articles alerted on the potential deleterious effects of COVID-19 on healthcare workers^6–9^. This specific group of the workforce was at a potentially higher risk of exposure to the virus^10,11^, as well as an increased workload. Physicians, researchers and the medical community cautioned from potential short- and long-term effects on healthcare personnel. Subsequently, with the rise of post-COVID cases, the risk that healthcare workers could suffer from post-COVID themselves became a real concern^12–15^, with a triple burden on this group of professionals: having to care for patients, having to cope with the pandemic in general, and having potential post-acute sequelae of the virus itself.

Compared to the general population, healthcare workers were shown to suffer from an increased prevalence of fatigue, headache, cognitive impairment, stress, burnout, insomnia, myalgia and arthralgia in SARS-CoV-2 positive and negative individuals^16^. Additionally, these symptoms were further increased in individuals suffering from post-COVID^12,16^. These concerning findings have been shown in transversal studies and related commentaries^12–14,16–18^, however longitudinal data showing the evolution and prolonged burden on healthcare workers are lacking so far.

At this stage, information on the evolution of the pandemic toll, the health status and well-being of healthcare workers is unknown. There are no data on the potential progression of post-COVID symptoms in SARS-CoV-2 positive individuals, as well as the potential worsening of overall health and well-being related outcomes in all healthcare workers (SARS-CoV-2 positive or negative).

To fill this gap, we conducted a prospective longitudinal analysis based on a cohort of healthcare professionals followed up to two years after the beginning of the pandemic. The objective of this study was to evaluate the potential progression of post-COVID symptoms in SARS-CoV-2 positive individuals, as well as the overall pandemic toll on SARS-CoV-2 positive and negative healthcare workers almost 2 years after the start of the pandemic.

## Results

### Overall participants

Out of the initial n = 3083 participants that had answered at baseline in July 2021^16^, n = 900 completed the follow-up in December 2021 (response rate 29.1%). Among participants, n = 298 (33.1%) were nurses, n = 155 (17.2%) were administrative staff, and n = 133 (14.8%) were physicians. The characteristics as well as distribution by department are shown in Table 1. Participants had a mean age of 46.4 [standard deviation, SD 10.5] years, with 62.9% between 40 and 59 years of age, and 70.1% were women. Overall, 46.7% individuals had no pre-existing comorbidities, 57.7% never smoked, and 88.1% considered themselves to be in good health. About 50% of individuals were doing no physical activity, and 39.1% were doing less than prior to the COVID-19 pandemic. Out of participants, 70% of participants were fully vaccinated (2 or more doses), n = 662 (73.5%) had consistently negative SARS-CoV-2 tests and n = 238 (26.4%) had at least one documented or self-reported positive SARS-CoV-2 test. For those tested positive, the median time from infection to baseline was 240 [interquartile range, IQR 226–265] days and the median time from infection to the follow-up was 407 [IQR 394–439] days. About 5% of individuals had a reinfection. The baseline characteristics of the n = 900 participants were not significantly different from the initial n = 3083 participants except for age. In this latter group, mean age was 43.8 [SD 11] years, 72.3% were women, 35.0% had one or more positive SARS-CoV-2 test, 65.1% were fully vaccinated and 50.1% did not have co-morbidities.

**Table 1:** Baseline characteristics of participants (n = 900)*

	Total (n = 900)	Negative or not tested (n = 662)	Positive (n = 238)	*P*-value
	N (%)	N (%)	N (%)	
Age (SD) in years	46.4 (10.5)	46.3 (10.4)	46.6 (10.9)	
Age categories				0.581
Below 40	256 (28.4)	185 (27.9)	71 (29.8)	
40–59	566 (62.9)	416 (62.8)	150 (63.0)	
60 and above	78 (8.7)	61 (9.2)	17 (7.1)	
Sex				0.333
Male	269 (29.9)	192 (29)	77 (32.4)	
Female	631 (70.1)	470 (71)	161 (67.6)	
Position				0.058
Nursing staff	298 (33.1)	205 (31.0)	93 (39.1)	
Physicians	133 (14.8)	105 (15.9)	28 (11.8)	
Administrative staff	155 (17.2)	118 (17.8)	37 (15.5)	
Other therapists and healthcare professionals	65 (7.2)	51 (7.7)	15 (5.9)	
Managers	38 (4.2)	23 (3.5)	15 (6.3)	
Other-ancillary services	211 (23.4)	160 (24.2)	51 (21.4)	
Department				0.053
Acute medicine	85 (9.5)	66 (10.0)	19 (8.0)	
Geriatrics	95 (10.6)	62 (9.4)	33 (13.9)	
Hospital administration and services	123 (13.7)	87 (13.2)	36 (15.1)	
Internal medicine	70 (7.8)	49 (7.4)	21 (8.8)	
Laboratory and diagnostics	71 (7.9)	53 (8.0)	18 (7.6)	
Neurosciences	47 (5.2)	42 (6.4)	5 (2.1)	
Obstetrics, gynecology and pediatrics	97 (10.8)	70 (10.6)	27 (11.3)	
Oncology	20 (2.2)	10 (1.5)	10 (4.2)	
Primary care	71 (7.9)	51 (7.7)	20 (8.4)	
Psychiatry	57 (6.4)	45 (6.8)	12 (5)	
Surgery	67 (7.5)	51 (7.7)	16 (6.7)	
Technical services	60 (6.7)	43 (6.5)	17 (7.1)	
Annex services	31 (3.5	27 (4.1)	4 (1.7)	
Other	3 (0.3)	3 (0.5)	0 (0.0)	
Smoking status				0.011
Never smoked	519 (57.7)	390 (58.9)	129 (54.2)	
Current smoker	141 (15.7)	114 (17.2)	27 (11.3)	
Ex-smoker	223 (24.7)	145 (21.9)	78 (32.8)	
Prefer not to answer	17 (1.9)	13 (2.0)	4 (1.7)	
Physical activity				0.076
None	441 (49.0)	330 (49.8)	111 (46.6)	
Less than before the COVID-19 pandemic	352 (39.1)	245 (37.0)	107 (45.0)	
Same as before the COVID-19 pandemic	105 (11.7)	85 (12.8)	20 (8.4)	
Prefer not to answer	2 (0.2)	2 (0.3)	0 (0.0)	
Vaccination status				< 0.001
Not or partially vaccinated	264 (29.7)	142 (21.7)	122 (51.9)	
Fully Vaccinated	626 (70.3)	513 (78.3)	113 (48.1)	
Self-rated health				0.183
Poor	107 (11.9)	73 (11.0)	34 (14.3)	
Good	793 (88.1)	589 (89.0)	204 (85.7)	
Symptoms at testing				< 0.001
Asymptomatic	295 (33.5)	275 (42.8)	22 (9.2)	
Symptomatic	378 (42.9)	203 (31.6)	175 (73.5)	
Pauci-symptomatic	206 (23.4)	164 (25.5)	42 (17.6)	
Prefer not to answer	1 (0.0)	0 (0.0)	1 (0.4)	
Reinfection	46 (5.1)	29 (4.4)	17 (7.1)	0.097
Hospitalization	97 (10.8)	76 (11.5)	21 (8.8)	0.429
Pre-existing comorbidities				
None	420 (46.7)	302 (46.1)	118 (52.4)	0.268
Obesity or overweight	115 (12.8)	79 (12.1)	36 (16.0)	0.201
Hypertension	56 (6.2)	35 (5.3)	21 (9.3)	0.052
Diabetes	15 (1.7)	9 (1.4)	6 (2.7)	0.229
Respiratory disease	22 (2.4)	16 (2.4)	6 (2.7)	0.928
Cardiovascular disease	12 (1.3)	9 (1.4)	3 (1.3)	0.910
Headache disorders	114 (12.7)	85 (13.9)	29 (12.9)	0.796
Sleep disorders	109 (12.1)	88 (13.4)	21 (9.3)	0.069
Cognitive disorders	31 (3.4)	18 (2.7)	13 (5.8)	0.046
Anxiety disorders	27 (3.0)	21 (3.2)	6 (2.7)	0.614
Depression	19 (2.1)	17 (2.6)	2 (0.9)	0.112
Chronic fatigue syndrome	33 (3.7)	19 (2.9)	14 (5.9)	0.033
Rheumatological disorders	56 (6.2)	40 (6.1)	16 (7.1)	0.707
Chronic pain or fibromyalgia	8 (0.9)	5 (0.8)	3 (1.3)	0.476

*Other healthcare professionals include physical therapy, occupational therapy, speech therapy, dentists and dietitians.

Other-ancillary services include all technical, laboratory and communication services.

Acute medicine department includes emergency care, intensive care, anesthesia and pharmacology.

Internal medicine department includes hospitalized patients in the general internal medicine wards, and subspecialties (outpatient and inpatient), except for oncology.

Primary care department includes the outpatient clinics and the SARS-CoV-2 testing and vaccination centers.

Fully vaccinated is defined as having received 2 or more doses of anti-SARS-CoV-2 vaccination.

Self-rated health is evaluated based on the SF-12 questionnaire^29^.

Only pre-existing comorbidities are included in this table.

### Symptoms prevalence and evolution

Overall, n = 489 (54.3%) of participants reported at least one symptom at baseline compared to n = 616 (68.4%) at follow-up. The main symptoms were fatigue, headache, insomnia, cognitive impairment, stress/burnout, pain, digestive symptoms, dyspnea and cough. The prevalence of each symptom in SARS-CoV-2 negative and positive individuals at baseline and follow-up are presented in Table 2, with an increase in the overall prevalence of all the listed symptoms and a differentially larger increase in the SARS-CoV-2 negative group. When fatigue was present, 62.8% of individuals reported severe fatigue at baseline as defined by the Chalder fatigue scale, compared to 75.4% of cases at follow-up. When insomnia was present, 46.9% of individuals reported mild insomnia on the insomnia severity index, 34.8% reported moderate insomnia, and 4.5% severe insomnia at baseline, compared to 49.7% mild insomnia, 37.9% moderate insomnia, and 1.3% severe insomnia at follow-up. Details are shown in Table 2.

**Table 2:** The overall prevalence of symptoms, functional impairment and scores of quality of life in healthcare workers (n = 900), and by SARS-CoV-2 infection status*

	Baseline			*P*-value	Follow-up			*P*-value
	Total (n = 900)	Negative or not tested (n = 662)	Positive (n = 238)		Total (n = 900)	Negative or not tested (n = 662)	Positive (n = 238)	
	N (%)	N (%)	N (%)		N (%)	N (%)	N (%)	
Number of symptoms				0.001				0.650
None	398 (44.2)	317 (47.9)	81 (34.0)		289 (32.1)	218 (32.9)	71 (29.8)	
1 symptom	101 (11.2)	70 (10.6)	31 (13.0)		90 (10)	63 (9.5)	27 (11.3)	
2 symptoms	104 (11.6)	77 (11.6)	27 (11.3)		119 (13.2)	87 (13.1)	32 (13.4)	
3 symptoms	79 (8.8)	55 (8.3)	24 (10.1)		95 (10.6)	74 (11.2)	21 (8.8)	
4 symptoms	57 (6.3)	42 (6.3)	15 (6.3)		82 (9.1)	63 (9.5)	19 (8)	
5–10 symptoms	140 (15.6)	91 (13.7)	49 (20.6)		184 (20.4)	129 (19.5)	55 (23.1)	
> = 11 symptoms	21 (2.3)	10 (1.5)	11 (4.6)		41 (4.6)	28 (4.2)	13 (5.5)	
Symptoms								
Fatigue	374 (41.6)	261 (39.4)	113 (47.5)	0.031	459 (51.0)	331 (50.0)	128 (53.8)	0.317
Headache	154 (17.1)	103 (15.6)	51 (21.4)	0.039	235 (26.1)	172 (26.0)	63 (26.5)	0.883
Insomnia	132 (14.7)	94 (14.2)	38 (16.0)	0.509	153 (17.0)	116 (17.5)	37 (15.5)	0.486
Cognitive impairment	104 (11.6)	54 (8.2)	50 (21.0)	< 0.001	117 (13.0)	67 (10.1)	50 (21.0)	< 0.001
Overall stress-burnout	183 (20.3)	134 (20.2)	49 (20.6)	0.909	262 (29.1)	186 (28.1)	76 (31.9)	0.264
Mental exhaustion/Burnout	109 (12.1)	73 (11.0)	36 (15.1)	0.096	152 (16.9)	102 (15.4)	50 (21.0)	0.048
Stress	94 (10.4)	71 (10.7)	23 (9.7)	0.646	150 (16.7)	110 (16.6)	40 (16.8)	0.946
Feelings of sadness	47 (5.2)	34 (5.1)	13 (5.5)	0.846	75 (8.3)	55 (8.3)	20 (8.4)	0.964
Anxiety	35 (3.9)	25 (3.8)	10 (4.2)	0.771	70 (7.8)	51 (7.7)	19 (8.0)	0.890
Pain	216 (24.0)	146 (22.1)	70 (29.4)	0.023	291 (32.3)	220 (33.2)	71 (29.8)	0.336
Arthralgia	90 (10.0)	56 (8.5)	34 (14.3)	0.010	108 (12.0)	77 (11.6)	31 (13.0)	0.570
Myalgia	85 (9.4)	57 (8.6)	28 (11.8)	0.154	137 (15.2)	106 (16)	31 (13.0)	0.271
Neck pain	66 (7.3)	44 (6.6)	22 (9.2)	0.187	106 (11.8)	81 (12.2)	25 (10.5)	0.477
Back pain	55 (6.1)	40 (6.0)	15 (6.3)	0.886	87 (9.7)	72 (10.9)	15 (6.3)	0.041
Digestive symptoms	74 (8.2)	58 (8.8)	16 (6.7)	0.326	106 (11.8)	81 (12.2)	25 (10.5)	0.477
Dyspnea	51 (5.7)	26 (3.9)	25 (10.5)	< 0.001	60 (6.7)	29 (4.4)	31 (13.0)	< 0.001
Cough	36 (4.0)	27 (4.1)	9 (3.8)	0.841	105 (11.7)	78 (11.8)	27 (11.3)	0.857
Chest pain	12 (1.3)	6 (0.9)	6 (2.5)	0.063	21 (2.3)	14 (2.1)	7 (2.9)	0.469
Palpitations	43 (4.8)	19 (2.9)	24 (10.1)	< 0.001	42 (4.7)	24 (3.6)	18 (7.6)	0.014
Loss or change in smell	61 (6.8)	16 (2.4)	45 (18.9)	< 0.001	59 (6.6)	28 (4.2)	31 (13.0)	< 0.001
Loss or change in taste	38 (4.2)	11 (1.7)	27 (11.3)	< 0.001	40 (4.4)	24 (3.6)	16 (6.7)	0.047
Loss of appetite	19 (2.1)	11 (1.7)	8 (3.4)	0.118	18 (2.0)	14 (2.1)	4 (1.7)	0.682
Throat pain	20 (2.2)	14 (2.1)	6 (2.5)	0.715	72 (8.0)	58 (8.8)	14 (5.9)	0.160
Rash	17 (1.9)	10 (1.5)	7 (2.9)	0.164	16 (1.8)	9 (1.4)	7 (2.9)	0.113
Hair loss	25 (2.8)	16 (2.4)	9 (3.8)	0.272	33 (3.7)	24 (3.6)	9 (3.8)	0.912
Functional impairment								
Overall	12.7 (11.8–13.7)	6.3 (5.9–6.8)	29.7 (27.7–31.7)	< 0.001	23.9 (22.3–25.5)	24.1 (22.3–26.0)	23.3 (20.1–26.7)	0.688
Professional domain	16.8 (15.7–18.0)	8.2 (7.7–8.6)	40.3 (38.2–42.4)	< 0.001	34.5 (32.7–36.4)	34.3 (32.2–36.4)	35.1 (31.4–38.9)	0.704
Family domain	18.0 (16.8–19.2)	8.7 (8.2–9.1)	43.2 (41.1–45.4)	< 0.001	37.0 (35.1–38.9)	36.9 (34.5–39.1)	37.2 (33.3–41.2)	0.882
Social domain	18.5 (17.2–19.7)	8.8 (8.4–9.3)	44.5 (42.4–46.6)	< 0.001	37.1 (35.2–39.0)	36.8 (34.6–39.0)	38.0 (34.1–41.9)	0.596
Quality of life								
Physical component score	50.9 (50.5–51.3)	51.4 (50.9–51.9)	49.4 (48.5–50.3)	< 0.001	49.9 (49.4–50.3)	50.1 (49.5–50.6)	49.2 (48.2–50.2)	0.125
Mental component score	41.4 (41.1–41.8)	41.3 (40.9–41.0)	41.7 (41.0–42.4)	0.325	40.5 (40.1–40.9)	40.5 (40.1–40.9)	40.3 (39.6–41.1)	0.641

*Functional impairment was calculated using the Sheehan disability scale^32^.

Functional impairment was adjusted for outcome for age, sex, profession within healthcare workers, SARS-CoV-2 infection status, and the following comorbidities only if pre-existing: obesity or overweight, hypertension, diabetes, respiratory disease, cardiovascular disease, headache disorders, cognitive disorders, sleep disorders, depression, anxiety, hypothyroidism, rheumatologic disease, anemia, chronic pain or fibromyalgia, chronic fatigue syndrome and irritable bowel syndrome.

Quality of life physical and mental component scores were calculated using the SF-12 survey instrument^29^.

**Figure 1:**
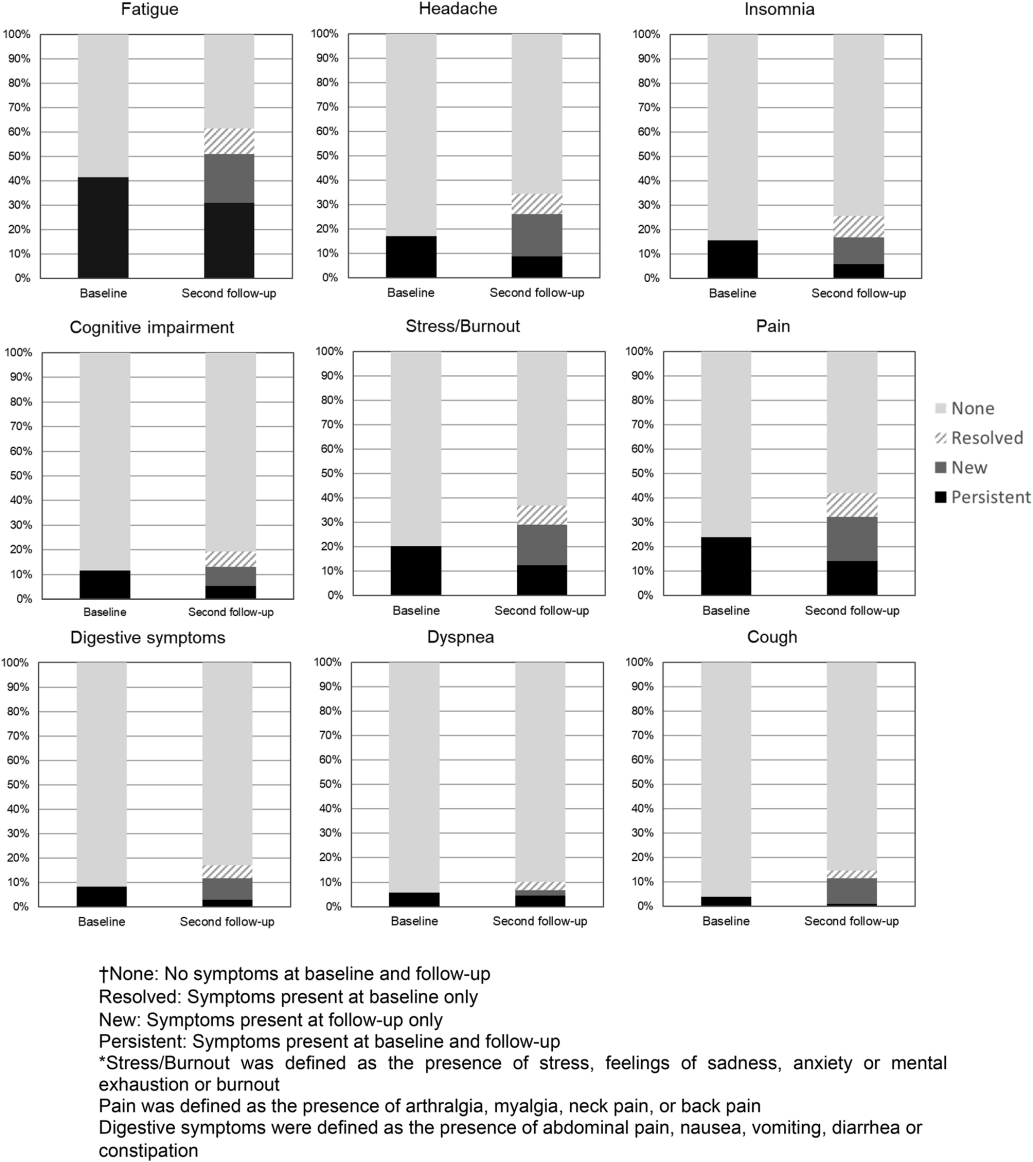
Symptoms evolution between baseline and follow-up (n = 900)†*.

The evolution of the main symptoms is reported in Fig. 1 and showed an increase in the prevalence of most symptoms, even after accounting for the resolution of symptoms in some individuals. At follow-up, more individuals reported fatigue (+ 9.4%), headache (+ 9.0%), insomnia (+ 2.3%), cognitive impairment (+ 1.4%), stress/burnout (+ 8.8%), pain (+ 8.3%), digestive symptoms (+ 3.6%), dyspnea (+ 1.0%), and cough (+ 7.7%) compared to baseline.

### Quality of life, functional capacity, and absenteeism

The SF-12 physical and mental health component scores for quality of life were both lower at follow-up compared to baseline. Individuals reported functional impairment in 12.7% of cases at baseline and 23.9% of cases at follow-up, with an increase in the prevalence of functional impairment in all three domains (professional, social, and family) in the SARS-CoV-2 negative individuals (Table 2).

Overall, n = 243 (36.7%) of SARS-CoV-2 negative individuals reported absence from work at baseline compared to n = 347 (52.4%) at the follow-up. At baseline, n = 38 (5.7%) of SARS-CoV-2 negative individuals had more than 10 days of absenteeism since the start of the pandemic compared to n = 149 (22.5%) at follow-up, and n = 104 (43.7%) of SARS-CoV-2 positive individuals compared to n = 117 (49.2%) at follow-up.

**Figure 2:**
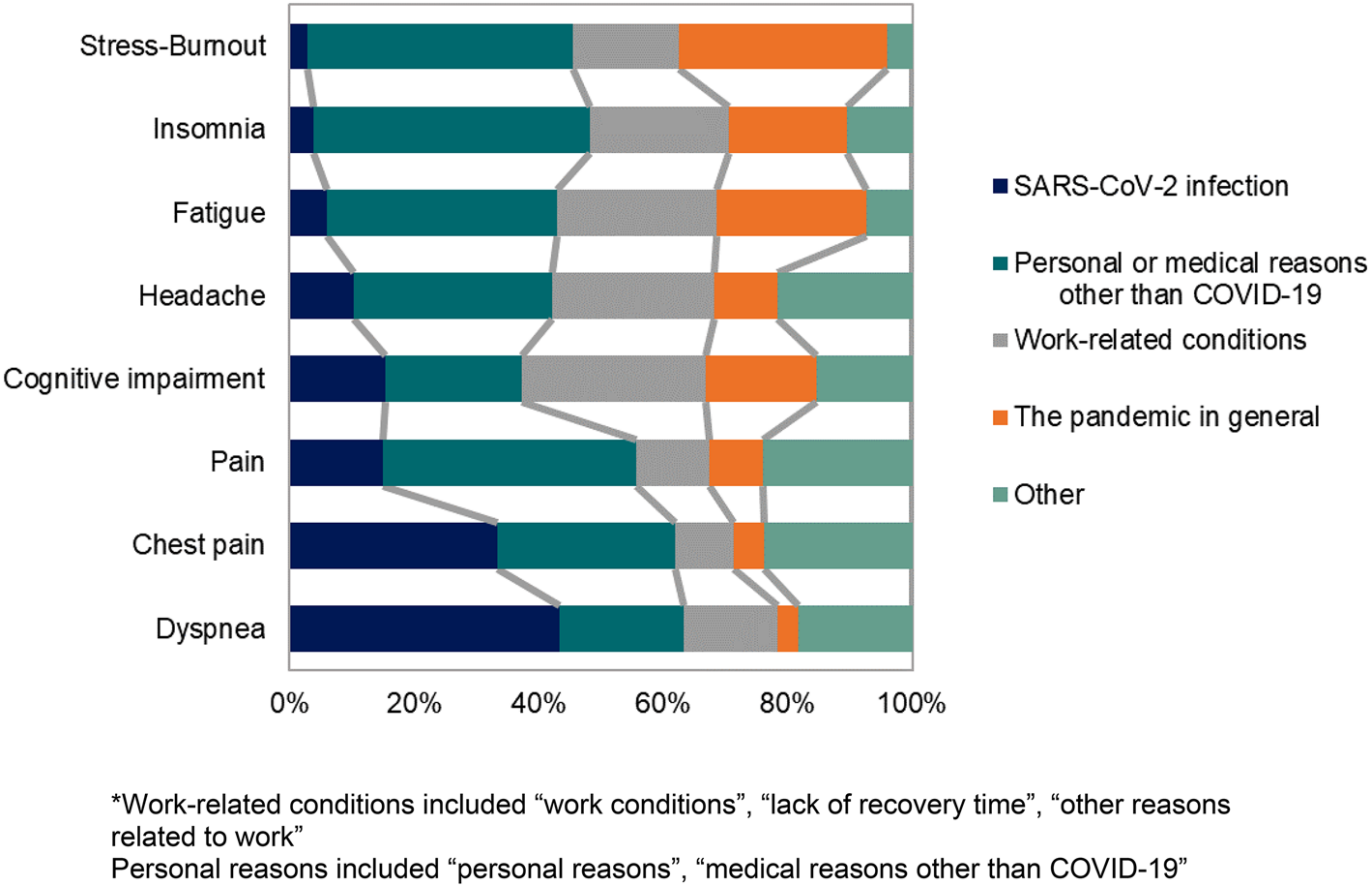
Self-reported reasons for symptoms by participants*.

Symptomatic participants reported personal reasons, the pandemic in general and lack of recovery time as some of the primary reasons for their symptoms. More SARS-CoV-2 negative individuals reported personal reasons and lack of recovery time as reasons for their symptoms, and infected participants reported SARS-CoV-2 infection as one of the reasons for their symptoms, however the overall distribution remained the same. There were no differences in the self-reported reasons by healthcare profession. Details are shown in Fig. 2. Supplement 2 shows in detail the self-reported reasons for symptoms and suggested solutions by participants.

Participants suggested the following solutions: more days off as the primary solution (22.4%), followed by benefits other than salary, including health insurance for example (18.6%), better work conditions (more personnel, more telework, better distribution of hours and schedules) in 13.7% of cases, better communication (10.9%), measures to favor work-life balance (9.1%), and a better salary in 3.1% of cases. There were no differences based on SARS-CoV-2 infection status nor healthcare profession.

## Discussion

This longitudinal study shows the evolution of symptoms in SARS-CoV-2 positive and negative individuals up to two years after the start of the COVID-19 pandemic. Healthcare workers have an increasing prevalence of symptoms including fatigue, headache, insomnia, cognitive impairment, stress, burnout, and pain with overall no improvement in symptoms among SARS-CoV-2 positive individuals, and a differentially larger increase in symptoms in SARS-CoV-2 negative individuals driving the increase in overall prevalence.

The prevalence of symptoms increased in healthcare workers compared to results shown previously^16^ and to the general population^19^. In a previous study using the same source population, results showed fatigue in 25.5% of healthcare workers, headache in 10.0%, insomnia in 6.2%, cognitive impairment in 7.9%, stress and burnout in 7.1% of cases^16^. Comparatively, individuals in the general population were shown to suffer less than healthcare workers^16,19^, and the current results show an even larger gap between the two groups. This underlines the differential impact of the pandemic on healthcare workers whether through direct effects (SARS-CoV-2 infection) or indirect effects (pandemic toll, work-related reasons).

SARS-CoV-2 positive individuals had more symptoms than SARS-CoV-2 negative individuals at baseline, and the prevalence of symptoms in SARS-CoV-2 positive individuals remained elevated at follow-up. SARS-CoV-2 positive individuals attributed their symptoms to personal reasons and the pandemic in general, and 19.7% of them attributed their absence from work to post-COVID symptoms (data not shown). Post-COVID condition remains a real concern for healthcare workers and the population in general, with the risk of post-acute sequelae increasing with reinfection^20^. Treatment options and up-to-date vaccination are some of the suggested solutions^20–24^, and this topic has now opened the page to post-acute infection syndromes in general^15^. Some of the postulated hypotheses so far are a dysregulation of the immune system, a persistent viral infection, or microclots^24^. A better understanding of the underlying mechanisms is needed with hopefully more and better solutions to come^23^.

SARS-CoV-2 negative individuals had a significantly larger increase in their symptoms between baseline and follow-up. This shows that those that were not infected might have suffered increasingly more from a work-related burden and the pandemic in general. Previous results from the same source population showed that 21.4% of SARS-CoV-2 negative individuals had fatigue, 7.8% headache, 5.3% insomnia, 4.6% cognitive impairment, and 6.3% stress/burnout^16^. Additionally, in another study, 3.1% of SARS-CoV-2 negative individuals in the general population suffered from fatigue at 12–16 months after the beginning of the pandemic, 1.7% suffered from headache, 2.7% insomnia, 2.5% cognitive impairment, and 1.4% suffered from stress/burnout^19^. While the results cannot be directly compared, there seems to be a higher prevalence of overall symptoms in healthcare workers compared to the general population confirming pre-pandemic studies showing high levels of fatigue and burnout in healthcare workers^25,26^, and potentially warning against an acceleration of this phenomenon with the COVID-19 pandemic.

When considering functional capacity, 12.7% of participants reported functional impairment at baseline compared to 23.9% at follow-up. This increase was mainly driven by a large increase in functional impairment in SARS-CoV-2 negative individuals (6.3% at baseline, 24.1% at follow-up), while the prevalence of functional impairment remained elevated in SARS-CoV-2 positive individuals (29.7% at baseline, compared to 23.3% at follow-up). The increase in functional impairment was seen in all domains of life (professional, social, and family). Similarly, the physical and mental component scores on the SF-12 quality of life scale showed a decrease in both domains, further underlining the impact of the COVID-19 pandemic on healthcare workers. Days of absenteeism, initially seen mainly in the SARS-CoV-2 positive group shifted at follow-up to include absenteeism in both SARS-CoV-2 positive and negative individuals. Participants reported personal reasons, the pandemic in general and the lack in recovery time as some of the primary reasons for their symptoms. This might be due to the added burden that healthcare workers had to endure, while some colleagues were absent for COVID-19 or other reasons. The extra burden and functional impairment need to be addressed, especially with increasing absenteeism, potentially transferring costs onto the remaining staff.

The high risk of burnout was mentioned early on during the pandemic^7–9^, and experts cautioned against this. Transversal studies looked into the prevalence of post-COVID symptoms^12–14,16^, as well as psychological distress in healthcare workers^13,14^ independently of SARS-CoV-2 infection, and experts sounded the alarm on the state of well-being of staff “Clinicians heal thyself”^18^. Related works showed that a potentially considerable proportion of healthcare workers were exposed to SARS-CoV-2, with an increased relative risk related to personal protective equipment, the workplace setting, contacts, and testing^14^. Healthcare workers who had even mild cases of COVID-19 were at risk of developing persistent symptoms^12^. In the study by Havervall et al., 15% of seropositive healthcare workers reported at least 1 moderate to severe symptom lasting for at least 8 months compared to 3% of seronegative healthcare workers (RR 4.4 [95% CI 2.9–6.7])^12^. Additionally, related works reported the potential impact of the pandemic on healthcare workers, with a high prevalence of depression, anxiety and post-traumatic stress disorder^27^. A systematic review and meta-analysis including 65 studies conducted across 21 countries between December 2019 and August 2020, showed a pooled 22.1% prevalence of anxiety, 21.7% prevalence of depression of 21.7%, and 21.5% prevalence of post-traumatic stress disorder (PTSD)^27^. Another systematic review and meta-analysis including 31 studies indicated a 30% prevalence of anxiety, 31.1% prevalence of depression, 31.4% prevalence of psycho-traumatic disorders, and 44.0% prevalence of sleep disorders^28^. This last study attempted to examine the effect of time suggesting an increase in the prevalence of sleep disorders with time. The study results were heterogenous and did not show other significant effects of time on the other outcomes^28^. In comparison, this present study looked into the longitudinal aspect of the evolution of symptoms in both SARS-CoV-2 positive and negative individuals, considering the effect of post-COVID condition. This present study showed an accelerated worsening of physical health, mental health, functional capacity and overall quality of life in healthcare workers. The longitudinal evolution and increase in the prevalence of symptoms were attributed to post-COVID condition as well as the differential impact of the pandemic on SARS-CoV-2 negative individuals. Of note, most studies examining the impact of the COVID-19 pandemic considered the early waves, and additional work was needed to show the protracted toll of the pandemic^18^.

Limitations include the self-reported nature of the follow-ups as well as the limited response rate. With a limited response rate and the nature of the follow-ups there is a potential risk of selection bias. Yet, at this stage, the information provided is valuable as no other data are available on the evolution of symptoms and the burden of the pandemic and post-COVID condition in healthcare workers. Additionally, the similarity of baseline characteristics between the participants in this longitudinal follow-up and the initial n = 3,083 participants who were invited to participate^16^, mitigates selection bias. Some calculations were underpowered when comparing symptoms in different groups at baseline and at follow-up. However, more power would have given a statistical significance that would not have been clinically relevant.

Healthcare workers are the backbone of the healthcare system. Their role in the lives of patients, the healthcare system and public health in general is essential. SARS-CoV-2 infection has brought acute absenteeism as well as post-COVID symptoms, and the COVID-19 pandemic has brought an extra burden of workload and stress on healthcare workers. Post-COVID caused absenteeism and might have transferred these costs on SARS-CoV-2 negative individuals, on top of the existing pandemic toll in general. Post-COVID is an opportunity to rethink post-acute infection syndromes in general^15^, and the COVID-19 pandemic should be the opportunity to reconsider the conditions of the healthcare workforce. Staff well-being should be an essential strategy at this stage. Looking into the details of absenteeism (trends per unit, department, position or other), and suggesting solution-oriented work schedules or predictability by building redundancy has now become an emergency to deal with post-COVID and the pandemic burden in general.

## Methods

### Ethical approval and consent to participate

Informed consent was obtained from all participants in this study. The study was approved by the Cantonal Research Ethics Commission of Geneva, Switzerland (Protocol no. 2021-00931). All methods were performed in accordance with the relevant guidelines and regulations.

### Participants and study setting

All staff of the Geneva University Hospitals (HUG) were invited for an online follow-up in July 2021 and then again in December 2021. The definition of healthcare workers included all hospital staff.

### Data collection

The questionnaire included questions about baseline characteristics, comorbidities, self-rated health, symptoms and evolution of symptoms since testing, current symptoms over the past two weeks. The 12-item short survey (SF-12) questionnaire^29^ was used to assess quality of life of healthcare workers. Self-rated health was assessed using the first question of the SF-12 questionnaire “How would you rate your health in general?” with answers including “excellent”, “very good”, “good”, “fair” and “poor”; answers were later categorized into two categories: 0 (poor to fair) and 1 (good to excellent). A physical component score (PCS) and mental component score (MCS) were calculated based on the answers to the SF-12 questionnaire, these scores generally have a mean of 50 (standard deviation of 10)^29^. A score of 50 or less on the PCS indicated a potential physical condition limiting the quality of life, and a score of 42 or less on the MCS indicated possible clinical depression^30,31^.

The Sheehan disability scale^32^ was used to assess functional capacity in the professional, social and family domains using a 10-point visual scale with 0 (no impairment at all), 1–3 (mild impairment), 4–6 (moderate impairment), 7–9 (marked impairment) and 10 (extreme impairment). A score of 5 or more in any of the three domains (professional, social or family) indicated functional impairment^32^. The Sheehan disability scale was also used to assess the days lost and days with reduced productivity due to functional impairment in the week preceding the survey. Additionally, the number of days with unscheduled absence from work was calculated and divided into categories “1–10 days”, “11–20 days”, “beyond 20 days”.

The Chalder fatigue scale^33^ was used to assess fatigue severity, using the 4-item Likert scale and the bimodal scoring scheme^34^. A score of ≥ 4 out of 11 on the bimodal scoring indicated severe fatigue. Individuals were also asked what they believed would have been the main reason for their fatigue with answers including: “SARS-CoV-2 infection”, “work conditions”, “lack of recovery time”, “other reasons related to work”, “the pandemic in general”, “medical reasons other than COVID-19”, “personal reasons”, “other”. Answers were then grouped into “SARS-CoV-2 infection”, “work-related reasons”, “the pandemic in general”, “personal or medical reasons other than COVID-19”, and “other”. This question was repeated for most symptoms including cognitive impairment, myalgia, arthralgia, headache, dizziness, insomnia, stress, burnout, anxiety, feelings of depression, chest pain, palpitations and dyspnea. Dyspnea was additionally assessed using the modified Medical Research Council (mMRC) scale^35^, and insomnia was assessed using the insomnia severity index (ISI)^36^. A Likert scale was used to assess the intensity of each symptom at the time of follow-up with self-reported options of “mild”, “moderate” or “severe”, and the frequency of each symptom in the two weeks preceding the follow-up with self-reported options of “never”, “rarely”, “often” or “always”.

As a more qualitative analysis, individuals were asked what they believed could reduce their stress or burnout symptoms with the following options that were not mutually exclusive: “better work conditions (better hours, more personnel)”, “support from the institution to favor a balance between the professional and personal life”, “better salary”, “better communication”, “more days off”, “benefits other than salary (health insurance, other)”, “no solution”, “prefer not to answer”, “other”. Escalating workload, inadequate support and communication have been shown to be reasons leading to healthcare burnout^37^.

The complete survey instrument is available in Supplement 1.

Age categories were defined as “below 40”, “40–59 years”, “60 years and above1” on the basis of previous studies suggesting that middle age may be a predictor of persistent symptoms^38^. Cognitive impairment was defined as the presence of self-reported difficulty concentrating or loss of memory. Pain was defined as the presence of arthralgia, myalgia, neck pain, back pain, or generalized pain. Stress/Burnout was defined as the presence of stress, feelings of sadness, anxiety or mental exhaustion or burnout. Functional impairment was defined as having mild, moderate or severe functional impairment using the Sheehan Disability Scale at the time of follow-up.

### Data analysis

Data was collected using REDCap v11.0.3 and analyzed using the statistical software Stata, version 16.0 (StataCorp). Descriptive analyses included percentages with comparisons using chi-square tests and Student’s t-test. Estimates of the prevalence of functional impairment were calculated using the logistic regression and the predict function, after adjusting this outcome for age, sex, profession within healthcare workers, SARS-CoV-2 infection status, and the following comorbidities only if pre-existing: obesity or overweight, hypertension, diabetes, respiratory disease, cardiovascular disease, headache disorders, cognitive disorders, sleep disorders, depression, anxiety, hypothyroidism, rheumatologic disease, anemia, chronic pain or fibromyalgia, chronic fatigue syndrome and irritable bowel syndrome, as based on previous studies on post-COVID^19^. A p-value of less than 0.05 was considered significant.

## Supplementary Information

Supplementary Information 1.

Supplementary Information 2.

## Data Availability

Our data are accessible to researchers upon reasonable request for data sharing to the corresponding author. This includes de-identified participant data or other additional related documents.
