# Supplementary material for: The longitudinal study of subjective wellbeing and absenteeism of healthcare workers considering post-COVID condition and the COVID-19 pandemic toll
Source: Sci Rep. 2023 Jul 4;13:10759. doi: 10.1038/s41598-023-37568-1 (PMC10319731; doi:10.1038/s41598-023-37568-1)
Supplement: Supplementary file 1 — Supplementary Information 1. [file 41598_2023_37568_MOESM1_ESM.docx]

| **Supplement 1. Survey instrument** | | |  | |  |
| --- | --- | --- | --- | --- | --- |
| Position | Administrative staff  Nursing personnel | | | | |
|  | Physicians  Other healthcare professionals (physical therapy, occupational therapy, speech therapy, dentists and dietitians) | | | | |
|  | Medical technicians  Technical services  Sanitation and restauration staff  Communication services | | | | |
| Department | Acute medicine  Geriatrics  Hospital administration and services  Internal medicine  Laboratory and diagnostics  Neurosciences  Obstetrics, gynecology and pediatrics  Oncology  Primary care  Psychiatry  Surgery  Technical services  Annex services  Other | | | | |
|  |  |  |  |  |  |
|  |  |  |  |  |  |
|  |  |  |  |  |  |
|  |  |  |  |  |  |
|  |  |  |  |  |  |
|  |  |  |  |  |  |
|  |  |  |  |  |  |
|  |  |  |  |  |  |
|  |  |  |  |  |  |
|  |  |  |  |  |  |
| Smoking status | I have never smoked  I am a current smoker  I am an ex-smoker  I prefer not to answer | | | | |
| Physical activity | I do not do any physical activity  I do less physical activity than prior to the COVID-19 pandemic  I do the same level of physical activity than prior to the COVID-19 pandemic  I prefer not to answer | | | | |
| Do you suffer from any of the following conditions (multiple answers possible) | None  Overweight  Sleep disorders  Migraine  Hypertension  Anxiety  Irritable bowel syndrome  Depression  Chronic fatigue syndrome  Respiratory disease  Other arthritic disorder (specify)  Tension headache  Tendinitis  Obesity  Anemia  Attention disorders  Memory disorders  Hypothyroidism  Other digestive disorder (specify)  Cardiovascular disease  Other type of headache (specify)  Diabetes  Chronic pain syndrome  Immunosuppression  Deep vein thrombosis  Dysmenorrhea  Hyperthyroidism  Rheumatoid arthritis  Other neurologic disorder (specify)  Fibromyalgia  Ulcerative colitis  Multiple sclerosis  Cancer  HIV  Other psychiatric disorder (specify)  Reactive arthritis  Renal disease  Ankylosing spondylitis  Crohn disease  Lupus  Sjogren disease  Cirrhosis | | | | |
| If condition checked, specify | [This condition] is new since my test date  I suffer from [this condition] since before my test date  Prefer not to answer | | | | |
| Did you have any nasopharyngeal swab (RT-PCR or antigenic test) positive for COVID-19? | Yes  No | | | | |
| If tested for SARS-CoV-2 | Did you have COVID-19 compatible symptoms at time of testing? | Yes, I had symptoms  Yes, but very few symptoms  No, I did not have symptoms  I prefer not to answer | | | |
|  | After your test date, how did your symptoms evolve? | I never had symptoms  My symptoms disappeared  My symptoms are fluctuating  My symptoms are constant  Other  I prefer not to answer | | | |
|  | Did you receive any of the following treatments in the first 21 days after your test date? (multiple answers possible):  None  Paracetamol  Non-steroidal anti-inflammatory medication  Antiviral (ritonavir, lopinavir, remdesivir etc.)  Dexamethasone, prednisone or other steroid  Monoclonal antibodies (Tocilizumab etc.)  Inhaled spray (Seretide®, Ventolin®, Symbicort®, Spiriva®, Atrovent®, Bricanyl®, Dospir®, etc.)  Nasal spray (Nasonex®, Rhinomer® etc.)  Hydroxychloroquine (Plaquenil®)  Ivermectin  Anticoagulation (Lovenox®, Arixtra® etc.)  Homeopathic treatment  Zinc  Vitamin C  Vitamin D  Other  I prefer not to answer | | | | |
| Have you had any serological testing for COVID-19 | Yes  No | | | | |
| Have you been vaccinated against SARS-CoV-2 | Yes  No  I prefer not to answer | | If yes, how many doses and date of each dose | |  |
| Which type of vaccine did you receive | Comirnaty® (BNT162b2) vaccine of Pfizer/BioNTech  (mRNA-1273) vaccine of Moderna  Vaxzevria (previously COVID-19 vaccine of Oxford/AstraZeneca)  Sinopharm BIBP vaccine of China National Pharmaceutical Group  Sputnik V vaccine of Gamaleya Research Institute of Epidemiology and Microbiology  Janssen Vaccine of Johnson&Johnson  I do not know  I prefer not to answer  Other | | | | |
| What was or has been the duration of symptoms since they started? | 0-10 days  11-20 days  21-30 days  1-2 months  2-3 months  3-4 months  4-5 months  5-6 months  6-7 months  8-9 months  9-10 months  10-11 months  11-12 months  More than 12 months  Do not know | | | | |
| Have you seen a physician or healthcare professional in relation to your symptoms?  (multiple answers possible) | Yes, my primary care physician  Yes, I have been to the emergency room  Yes, I have seen another specialist, physician or healthcare professional (specify)  No | | If yes, how many times for each answer and is this a new follow-up since your test date? | |  |
| Have you been hospitalized since the start of the pandemic? | Yes  No  I prefer not to answer | | If yes, was the hospitalization related to COVID-19 and date of hospitalization | |  |
| In the past 2 weeks, which of the following symptoms have you experienced, even if fluctuating? (multiple answers possible) | Fatigue  Headache  Change in smell  Mental exhaustion  Myalgia  Dyspnea  Difficulty concentrating  Insomnia  Stress  Change in taste  Loss of memory  Paresthesia  Arthralgia  Neck pain  Anxiety  Cough  Sadness  Loss of smell  Palpitations  Dizziness  Back pain  Throat pain  Diarrhea  Nausea  Abdominal pain  Loss of taste  Hair loss  Lack of equilibrium  Lack of appetite  Chest pain  Constipation  Generalized pain  Fever > 38 C  Rash  Malaise  Vomiting  Toe pain or redness  Finger pain or redness  Other  I prefer not to answer | | | | |
| If symptom checked, did you have this symptom regularly (most days) prior to the pandemic? | Yes  No  I prefer not to answer | | | | |
| Current symptom intensity | Mild  Moderate  Severe | | | | |
| Current symptom frequency | Never  Rarely  Often  Always | | | | |
| If fatigue, specify | Normal activity, no limitations in daily activity  Limited activity but capable to do light work (office work, cleaning)  Limited activity but <50% in bed during the day  >50% in bed, but not bedbound  Bedbound | | | | |
| If fatigue, | Do you have problems with tiredness? | | Less than usual  No more than usual  More than usual  Much more than usual | |  |
|  | Do you need to rest more? | | Less than usual  No more than usual  More than usual  Much more than usual | |  |
|  | Do you feel sleepy or drowsy? | | Less than usual  No more than usual  More than usual  Much more than usual | |  |
|  | Do you have problems starting things? | | Less than usual  No more than usual  More than usual  Much more than usual | |  |
|  | Do you lack energy? | | Less than usual  No more than usual  More than usual  Much more than usual | |  |
|  | Do you have less strength in your muscles? | | Less than usual  No more than usual  More than usual  Much more than usual | |  |
|  | Do you feel weak? | | Less than usual  No more than usual  More than usual  Much more than usual | |  |
|  | Do you have difficulty concentrating? | | Less than usual  No more than usual  More than usual  Much more than usual | |  |
|  | Do you make slips of the tongue when speaking? | | Less than usual  No more than usual  More than usual  Much more than usual | |  |
|  | Do you find it more difficult to find the right word? | | Less than usual  No more than usual  More than usual  Much more than usual | |  |
|  | Is your memory as good as usual? | | Less than usual  No more than usual  More than usual  Much more than usual | |  |
| If dyspnea, specify | Dyspnea only with strenuous exercise  Dyspnea when hurrying or walking up a slight hill  Has to stop for breath when walking at own pace on flat surface  Stops for breath after walking 90 meters or after a few minutes  Too breathless to leave house or breathless when dressing | | |  | |
| If insomnia, specify (over the past 2 weeks) | Do you have difficulty falling asleep? | | None  Mild  Moderate  Severe  Very severe | |  |
|  | Do you have difficulty staying asleep? | | None  Mild  Moderate  Severe  Very severe | |  |
|  | Do you have problems waking up too early? | | None  Mild  Moderate  Severe  Very severe | |  |
|  | How satisfied/dissatisfied are you with your current sleep pattern? | | Very satisfied  Satisfied  Moderately satisfied  Dissatisfied  Very dissatisfied | |  |
|  | How noticeable to others do you think your sleep problem is in terms of impairing the quality of your life? | | Not at all noticeable  A little  Somewhat noticeable  Very much noticeable | |  |
|  | How worried/distressed are you about your current sleep problem? | | Not at all worried  A little  Somewhat worried  Very much worried | |  |
|  | To what extent do you consider your sleep problem to interfere with your daily functioning currently? | | Not at all interfering  A little  Somewhat interfering  Very much interfering | |  |
| If symptom checked, specify: | Your symptoms have disrupted your work/school work | | Scale:  0 Not at all – 10 Extremely | |  |
|  | Your symptoms have disrupted your social life/leisure activities | | Scale:  0 Not at all – 10 Extremely | |  |
|  | Your symptoms have disrupted your family life/home responsibilities | | Scale:  0 Not at all – 10 Extremely | |  |
|  | How many days in the last week did your symptoms cause you to miss work or leave you unable to carry out your normal daily responsibilities? | | | | |
|  | How many days in the last week did you feel so impaired by your symptoms that even though you went to work, your productivity was reduced? | | | | |
| In general, would you say your health prior to the test date was | Excellent, very good, good, fair, poor | |  | |  |
| In general, would you say your health is currently | Excellent, very good, good, fair, poor | |  | |  |
| The following questions are about activities you might do during a typical day. Does your health now limit you in these activities? If so, how much? | Moderate activities such as moving a table, pushing a vacuum cleaner, bowling, or playing golf | | Yes, limited a lot  Yes, limited a little  No, not limited at all | |  |
|  | Climbing several flights of stairs | | Yes, limited a lot  Yes, limited a little  No, not limited at all | |  |
| During the past 4 weeks, have you had any of the following problems with your work or other regular daily activities as a result of your physical health? | Accomplished less than you would like | | Yes  No | |  |
|  | Were limited in the kind of work or other activities | | Yes  No | |  |
| During the past 4 weeks, have you had any of the following problems with your work or other regular daily activities as a result of any emotional problems (such as feeling depressed or anxious)? | Accomplished less than you would like | | Yes  No | |  |
|  | Did work or activities less carefully than usual | | Yes  No | |  |
| During the past 4 weeks, how much did pain interfere with your normal work (including work outside the home and housework)? | Not at all  A little bit  Moderately  Quite a bit  Extremely | |  | |  |
| These questions are about how you have been feeling during the past 4 weeks.  For each question, please give the one answer that comes closest to the way you have been feeling.  How much of the time during the past 4 weeks… | Have you felt calm and peaceful? | | All of the time  Most of the time  A good bit of the time  Some of the time  A little of the time  None of the time | |  |
|  | Did you have a lot of energy? | | All of the time  Most of the time  A good bit of the time  Some of the time  A little of the time  None of the time | |  |
|  | Have you felt calm and peaceful? | | All of the time  Most of the time  A good bit of the time  Some of the time  A little of the time  None of the time | |  |
|  | Have you felt down-hearted and blue? | | All of the time  Most of the time  A good bit of the time  Some of the time  A little of the time  None of the time | |  |
| During the past 4 weeks, how much of the time has your physical health or emotional problems interfered with your social activities (like visiting friends, relatives, etc.)? | All of the time  Most of the time  A good bit of the time  Some of the time  A little of the time  None of the time | |  | |  |
