# Supplementary material for: The longitudinal study of subjective wellbeing and absenteeism of healthcare workers considering post-COVID condition and the COVID-19 pandemic toll
Source: Sci Rep. 2023 Jul 4;13:10759. doi: 10.1038/s41598-023-37568-1 (PMC10319731; doi:10.1038/s41598-023-37568-1)
Supplement: Supplementary file 2 — Supplementary Information 2. [file 41598_2023_37568_MOESM2_ESM.docx]

**Supplement 2. Self-reported reasons for symptoms and suggested solutions by participants (n=900)**

|  | **Total (n=900)** | **Negative (n=662)** | **Positive (n=238)** | **P-value** |
| --- | --- | --- | --- | --- |
| **Self-reported reasons for:** | N(%) | N(%) | N(%) |  |
| **Fatigue** |  |  |  | 0.001 |
| SARS-CoV-2 infection | 27(5.9) | 10(3.0) | 17(13.1) |  |
| Work conditions | 50(10.9) | 37(11.1) | 13(10.0) |  |
| Lack of recovery time | 57(12.4) | 34(10.3) | 23(17.7) |  |
| Other professional reasons | 11(2.4) | 6(1.8) | 5(3.8) |  |
| The pandemic in general | 111(24.1) | 85(25.7) | 26(20) |  |
| Medical reason other than COVID-19 | 31(6.7) | 23(6.9) | 8(6.2) |  |
| Personal reasons | 140(30.4) | 110(33.2) | 30(23.1) |  |
| Other | 34(7.4) | 26(7.9) | 8(6.2) |  |
| **Cognitive impairment** |  |  |  | 0.007 |
| SARS-CoV-2 infection | 18(15.3) | 5(7.5) | 13(25.5) |  |
| Work conditions | 2(1.7) | 0(0) | 2(3.9) |  |
| Lack of recovery time | 28(23.7) | 18(26.9) | 10(19.6) |  |
| Other professional reasons | 5(4.2) | 4(6) | 1(2) |  |
| The pandemic in general | 21(17.8) | 12(17.9) | 9(17.6) |  |
| Medical reason other than COVID-19 | 5(4.2) | 2(3) | 3(5.9) |  |
| Personal reasons | 21(17.8) | 18(26.9) | 3(5.9) |  |
| Other | 18(15.3) | 8(11.9) | 10(19.6) |  |
| **Headache** |  |  |  | 0.001 |
| SARS-CoV-2 infection | 24(10.2) | 8(4.7) | 16(25.4) |  |
| Work conditions | 15(6.4) | 10(5.8) | 5(7.9) |  |
| Lack of recovery time | 40(17) | 30(17.4) | 10(15.9) |  |
| Other professional reasons | 6(2.6) | 6(3.5) | 0(0) |  |
| The pandemic in general | 24(10.2) | 20(11.6) | 4(6.3) |  |
| Medical reason other than COVID-19 | 41(17.4) | 32(18.6) | 9(14.3) |  |
| Personal reasons | 34(14.5) | 28(16.3) | 6(9.5) |  |
| Other | 51(21.7) | 38(22.1) | 13(20.6) |  |
| **Insomnia** |  |  |  | 0.008 |
| SARS-CoV-2 infection | 6(3.9) | 1(0.9) | 5(13.5) |  |
| Work conditions | 7(4.6) | 4(3.4) | 3(8.1) |  |
| Lack of recovery time | 16(10.5) | 12(10.3) | 4(10.8) |  |
| Other professional reasons | 11(7.2) | 8(6.9) | 3(8.1) |  |
| The pandemic in general | 29(19) | 23(19.8) | 6(16.2) |  |
| Medical reason other than COVID-19 | 5(3.3) | 5(4.3) | 0(0) |  |
| Personal reasons | 63(41.2) | 53(45.7) | 10(27.0) |  |
| Other | 16(10.5) | 10(8.6) | 6(16.2) |  |
| **Stress-burnout** |  |  |  | 0.230 |
| SARS-CoV-2 infection | 7(2.8) | 2(1.1) | 5(6.9) |  |
| Work conditions | 14(5.7) | 9(5.1) | 5(7.0) |  |
| Lack of recovery time | 14(5.7) | 11(6.3) | 3(4.2) |  |
| Other professional reasons | 14(5.7) | 12(6.9) | 2(2.8) |  |
| The pandemic in general | 82(33.3) | 54(31) | 28(38.9) |  |
| Medical reason other than COVID-19 | 13(5.3) | 9(5.2) | 4(5.6) |  |
| Personal reasons | 92(37.4) | 70(40.2) | 22(30.6) |  |
| Other | 10(4.1) | 7(4) | 3(4.2) |  |
| **Pain** |  |  |  | 0.001 |
| SARS-CoV-2 infection | 33(14.9) | 13(8.1) | 20(33.3) |  |
| Work conditions | 9(4.1) | 6/3.7) | 3(5.0) |  |
| Lack of recovery time | 14(6.3) | 10(6.2) | 4(6.7) |  |
| Other professional reasons | 3(1.4) | 3(1.9) | 0(0) |  |
| The pandemic in general | 19(8.6) | 15(9.3) | 4(6.7) |  |
| Medical reason other than COVID-19 | 49(22.2) | 36(22.4) | 13(21.7) |  |
| Personal reasons | 41(18.6) | 36(22.4) | 5(8.3) |  |
| Other | 53(24) | 42(26.1) | 11(18.3) |  |
| **Dyspnea** |  |  |  | 0.013 |
| SARS-CoV-2 infection | 26(43.3) | 7(24.1) | 19(61.3) |  |
| Work conditions | 0(0.0) | 0(0.0) | 0(0.0) |  |
| Lack of recovery time | 9(15) | 3(10.3) | 6(19.4) |  |
| Other professional reasons | 0(0.0) | 0(0.0) | 0(0.0) |  |
| The pandemic in general | 2(3.3) | 2(6.9) | 0(0) |  |
| Medical reason other than COVID-19 | 10(16.7) | 8(27.6) | 2(6.5) |  |
| Personal reasons | 2(3.3) | 1(3.4) | 1(3.2) |  |
| Other | 11(18.3) | 8(27.6) | 3(9.7) |  |
| **Chest pain** |  |  |  | 0.595 |
| SARS-CoV-2 infection | 7(33.3) | 3(21.4) | 4(57.1) |  |
| Work conditions | 0(0.0) | 0(0.0) | 0(0.0) |  |
| Lack of recovery time | 2(9.5) | 2(14.3) | 0(0.0) |  |
| Other professional reasons | 0(0.0) | 0(0.0) | 0(0.0) |  |
| The pandemic in general | 1(4.8) | 1(7.1) | 0(0.0) |  |
| Medical reason other than COVID-19 | 3(14.3) | 2(14.3) | 1(14.3) |  |
| Personal reasons | 3(14.3) | 2(14.3) | 1(14.3) |  |
| Other | 5(23.8) | 4(28.6) | 1(14.3) |  |
| **Solutions** |  |  |  | 0.182 |
| More days off | 202(22.4) | 137(20.7) | 65(27.3) |  |
| More benefits other than salary | 167(18.6) | 131(19.8) | 36(15.1) |  |
| Better work conditions (hours, personnel, telework) | 123(13.7) | 94(14.2) | 29(12.2) |  |
| Better communication | 98(10.9) | 74(11.2) | 24(10.1) |  |
| Favor work-life balance | 82(9.1) | 62(9.4) | 20(8.4) |  |
| Better salary | 28(3.1) | 24(3.6) | 4(1.7) |  |
| None | 19(2.1) | 15(2.3) | 4(1.7) |  |
| Prefer not to answer | 28(3.1) | 21(3.2) | 7(2.9) |  |
| Do not know | 134(14.9) | 89(13.4) | 45(18.9) |  |
| Other | 19(2.1) | 15(2.3) | 4(1.7) |  |
